# Characterization of Mechanical, Electrical and Thermal Properties of Bismaleimide Resins Based on Different Branched Structures

**DOI:** 10.3390/polym15030592

**Published:** 2023-01-24

**Authors:** Haihui Cai, Jiahao Shi, Xiaorui Zhang, Zhou Yang, Ling Weng, Qingye Wang, Shaohui Yan, Lida Yu, Junlong Yang

**Affiliations:** 1 School of Materials Science and Engineering, Harbin University of Science and Technology, Harbin 150040, China; 2 Harbin Institute of Large Electrical Machinery, Harbin 150040, China; 3 State Key Laboratory of Hydropower Equipment, Harbin 150040, China; 4 Harbin Electric Machinery Company Limited, Harbin 150040, China

**Keywords:** BMI resin, branched structure, toughness, flexural modulus, electrical properties

## Abstract

Bismaleimide (BMI) resin is an excellent performance resin, mainly due to its resistance to the effect of heat and its insulating properties. However, its lack of toughness as a cured product hampers its application in printed circuit boards (PCBs). Herein, a branched structure via Michael addition was introduced to a BMI system to reinforce its toughness. Compared with a pure BMI sample, the flexural strength of the modified BMI was enhanced, and its maximum value of 189 MPa increased by 216%. The flexural modulus of the cured sample reached 5.2 GPa. Using a scanning electron microscope, the fracture surfaces of BMI samples and a transition from brittle fracture to ductile fracture were observed. Furthermore, both the dielectric constant and the dielectric loss of the cured resin decreased. The breakdown field strength was raised to 37.8 kV/mm and the volume resistivity was improved to varying degrees. Consequently, the resulting modified BMI resin has the potential for wide application in high-frequency and low-dielectric resin substrates, and the modified BMI resin with a structure including three different diamines can meet the needs of various applications.

## 1. Introduction

As one of the core components of electronic equipment, the printed circuit board (PCB) undergoes an enormous loss of energy in the transmission process, which renders it unable to satisfy the requirements of transmitting high-frequency and high-speed communication signals [1,2,3,4]. Bismaleimide (BMI) resin is a class of high-performance polymers with a low dielectric constant, a low dielectric loss and heat resistance. However, standard BMI resins exhibit brittleness and low mechanical performance owing to their high cross-linking density (for example, a rigid network in a cured BMI resin system) [5,6,7,8]. These shortcomings greatly limit their application in the electronics field. Thus, it is essential to toughen BMI resin.

In order to toughen modified BMI resin, many studies around the world have been trying to obtain good performance. One of the most commonly used methods is via the impurity of the inorganic fillers, such as nano-SiO_2_ [9,10], carbon nanotube (CNT) [11,12,13], and graphene [14,15,16], in the resin system. Nanoparticles facilely agglomerate and disperse with difficulty into the resin system; the result of nanoparticles’ agglomeration is a deterioration of mechanical properties, without a toughing effect [17,18]. In addition, as the second phase, introducing rubber [19,20] thermoplastic polymer [21,22,23] into a BMI resin system is an effective toughening method; however, it results in a higher viscosity resin system. Wang et al. [24] fabricated a novel BMI resin system using BMPP/BTM/DABPA with vinyl-terminated butadiene acrylonitrile (VTBN). The result indicated that an increased K_IC_ in this novel BMI resin system inevitably has a substantial negative impact on its thermal resistance. Adding thermoplastic efficiently united the expected advantages of thermoplastic resin and thermosetting resin, which compensated for the defect that the heat resistance of rubber is depressed after toughening. Liu et al. [25] incorporated polyethersulfone into a BMI resin system, and the glass transition temperature (T_g_) increased slightly. However, whether using rubber or thermoplastic, physical mixing inevitably increases the viscosity in a resin system, which significantly deteriorates its processability. Therefore, physical mixing might not be an effective solution to reinforce BMI resin.

A common method of modifying BMI is copolymerization with a diamine. Through Michael addition, BMI and diamine form a flexible chain. The distance between the two functional groups increases, and the cross-linking density of the cured resin decreases [26,27,28]. The modified resin shows enhanced energy absorption capacity, thereby improving its mechanical performance. Another modification method is synthesizing BMI resin with a branched structure by designing the molecular structure, which can improve the mechanical strength of thermosetting polymer [29,30,31,32]. Jiang et al. [33] designed a novel liquid multi-maleimide branched polysiloxane (PMI-HSi). PMI-HSi in varying amounts was admixed with 4, 4’-bismaleimidophenyl methane (BDM) and 2, 2’-diallyl bisphenol A (DBA) into a series of curing samples. The study stated that this clearly improved toughness without sacrificing thermal resistance. Lee et al. [34] presented an aliphatic-aromatic copolyimide using diamine with a branched structure. The branched bulky aliphatic units broadened the free volume, effectively reducing the dipole moment density and achieving a low-dielectric with dielectric constant and dielectric loss. Nevertheless, few studies have reported a combination of these two methods, and few scholars pay attention to the performance of the modulus. In this case, it is necessary to toughen BMI resins and discuss the change in modulus and dielectric properties.

**Figure 1:**
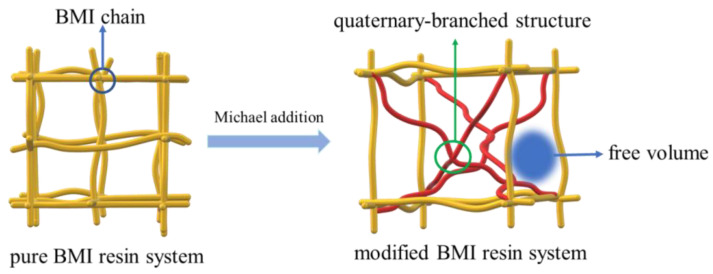
Cured network structure of modified BMI.

In this study, a branched structure was synthesized to toughen BMI resin through the Michael addition, inducing diamine into a BMI cross-linking network. The cured network BMI resin system is shown in Figure 1. The modified BMI resin system formed a branched structure, which introduced large free volume and reduced the density of the curing sample. Consequently, it could reduce the density of the curing material and improve the toughness of the BMI. At the same time, the rigid group contained in diamine also affected the mechanical properties of the product, the dipole moment density was significantly reduced, and both the dielectric constant and the dielectric loss reached a low value. Significantly, these results indicate that modified BMI resin has broad application prospects in high-frequency and low-dielectric resin substrates. At the same time, BMI resin modified by diamines with different structures can expand its application in the electronics field.

## 2. Experimental Method

### 2.1. Materials

Hubei Jinleda Chemical Co., Ltd. (Hubei, China) supplied 2,2’-Diallyl bisphenol A (BBA) and bismaleimide (BMI). Additionally, 4,4’-Oxydianiline (ODA), 9-9’Bis(4-aminophenyl)fluorene (FDA) and 2,2’-Dimethyl-[1,1’-Biphenyl]-4,4’-Diamine (MT) were purchased from Chinatech (Tianjin, China) Chemical Co., Ltd. All compounds were used without further treatment.

### 2.2. Sample Preparation

**Figure 2:**
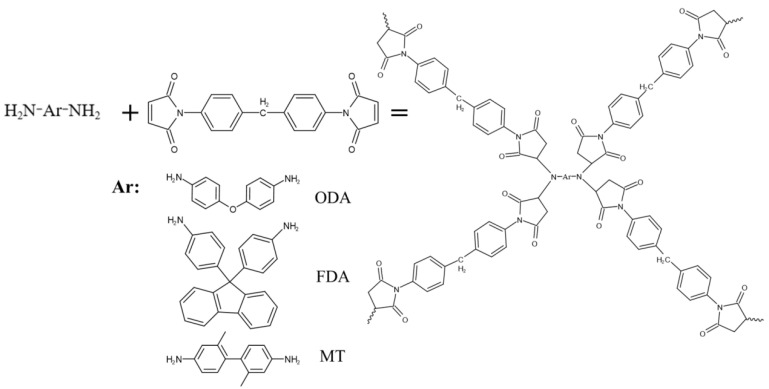
Reaction scheme for modified BMI resin.

**Figure 3:**
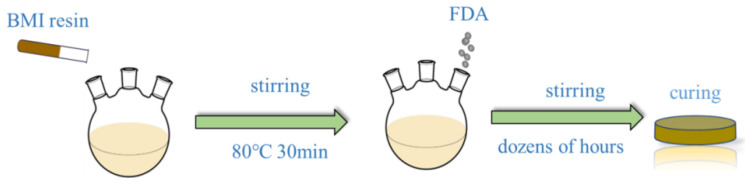
Reaction procedure for modified BMI resin.

A branched structure polymer was assembled via Michael addition with diamine and BMI resin, and the synthesis of BMI resin with diamine (BMI-NH_2_) has been previously described. The detailed formulations for the preparation are shown in Table 1. BMI resin was added to a flask at 80 ℃ under stirring for 30 min to preheat the BMI resin. Subsequently, FDA was added (FDA and BMI resin molar ratio 1:4), and then the reaction solution was stirred at 80 ℃ for dozens of hours. After a cooling treatment, the preheated mold was filled with the mixed resin for curing in steps: 150 ℃ for 1 h, 180 ℃ for 2 h, 200 ℃ for 2 h, and 220 ℃ for 1 h. After the oven was turned off and slowly cooled to room temperature, the cured resin samples were polished to a proper size for testing. The reaction scheme and the reaction procedure for the sample are depicted in Figure 2 and Figure 3.

### 2.3. Performance Testing

Mechanic properties: The flexural strength of the samples was tested using the Instron universal testing machine of TY8000-A (Tian Yuan Test Instrument, Jiangsu, China) at a speed of 10 mm·min^-1^. The sample specification was prepared as 100 mm × 10 mm × 5 mm. In order to avoid the deviation of results caused by uncertain factors, each sample was tested ten times. Sample points with obvious errors were removed, and the average flexural strength was obtained.

Scanning electron microscope (SEM): The JSM-7500F (Hitachi, Japan) was used to scan the samples’ surfaces. To obtain the surface morphology, the accelerating voltage of the device was adjusted to 3 kV.

Dynamic thermomechanical analysis (DMA): DMA results of samples were obtained using a TA Q800 dynamic mechanical analyzer manufactured by the TA Instrument Company (New Castle, DE, USA). The three-point bending pattern of the machine was used throughout the testing process with a 3 ℃/min temperature increase. The temperature ranged from 50 ℃ to 300 ℃. The sized samples (40 mm × 0.9 mm × 7 mm) were scanned at a vibration frequency of 1 Hz. Each diamine sample was tested once.

Insulate properties: The volume resistivity of samples was tested using EST121 (Digital Technology Co., Ltd. Beijing Hengrui Xinda, Beijing, China) with a measurement voltage of 1000 v. Five samples of each diamine, sized 100 mm × 100 mm × 1 mm, were tested. The electrical puncture strengths of samples were studied using the HT-100 breakdown voltage tester (Guilin Institute of Electrical Appliances, Guilin, China) in an oil bath environment; ten test sites were selected on each diamine sample. Dielectric constant and dielectric loss were evaluated using the Concept 80 Dielectric Spectrometer (Montabaur, Germany) in the frequency range of 10–10^7^ Hz; the samples were 30 mm × 30 mm × 1 mm. Tests were performed at room temperature, and each diamine sample was tested once.

Thermogravimetric analysis (TGA): The Pyris 1 TGA (PerkinElmer, Waltham, MA, USA) machine performed a complicated thermogravimetric analysis. The heating rate was 5 ℃/min with a range from 100 ℃ to 700 ℃, and the flow rate of purified nitrogen gas was 20 mL/min. The sample’s mass was 100 mg.

## 3. Results and Discussion

### 3.1. Mechanical Properties

**Figure 4:**
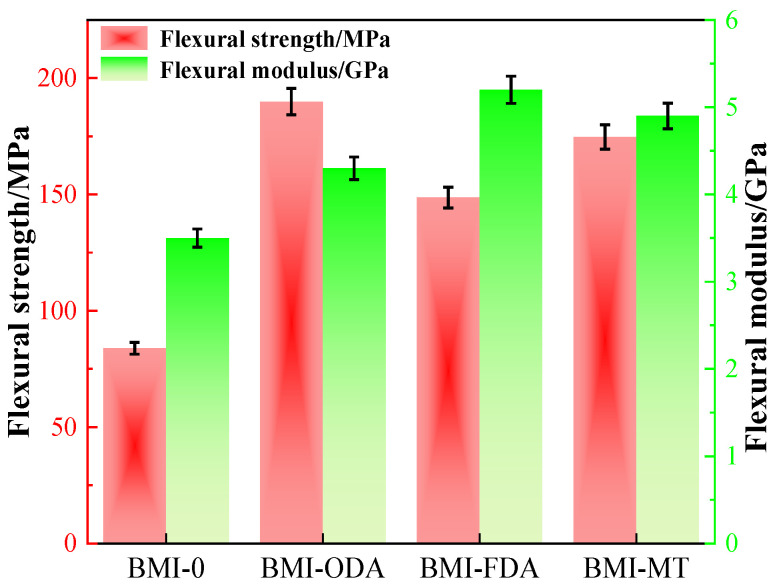
Mechanical properties of columns of BMI-NH_2_.

Figure 4 shows the mechanical properties of BMI-NH_2_. It is obvious that the flexural strength of BMI resin was modified by different types of diamines. The flexural strength of the pure BMI sample was 83.9 MPa, but the flexural strength of the sample after the Michael addition reaction reached an excellent value of 189.9 MPa using ODA, an increase of 226%. No matter what type of diamine was used, the flexural strength of the sample was improved, because the quaternary branching structure via Michael addition provided free volume without degrading the cross-linking intensity. It effectually reduced stress transfer received via external force, so that the samples showed preferable toughness [35].

Diamines ODA, MT and FDA have different molecular structures. As shown in Figure 2, ODA has a flexible segment with an ether bond. It can produce deformation under external force to obtain a toughening effect. FDA contains a fluorenyl, a rigid ring, and increases the cross-linking density per unit volume. It caused the flexible strength to be lower than ODA did. MT possesses a biphenylyl structure, similar to a rod structure, less rigid than FDA and less flexible than ODA; its bending strength lies in the middle between FDA and ODA.

Modulus is the ability of a material to resist deformation. As seen in the figure, the flexural modulus of BMI-FDA was 5.2 GPa, which was 1.5 times that of the pure BMI sample (3.5 GPa). This was consistent with our previous analysis. FDA contains a rigid group and had a more obvious ability to resist deformation. Therefore, it was harder to deform it under external forces; the value of BMI-FDA was the best.

**Figure 5:**
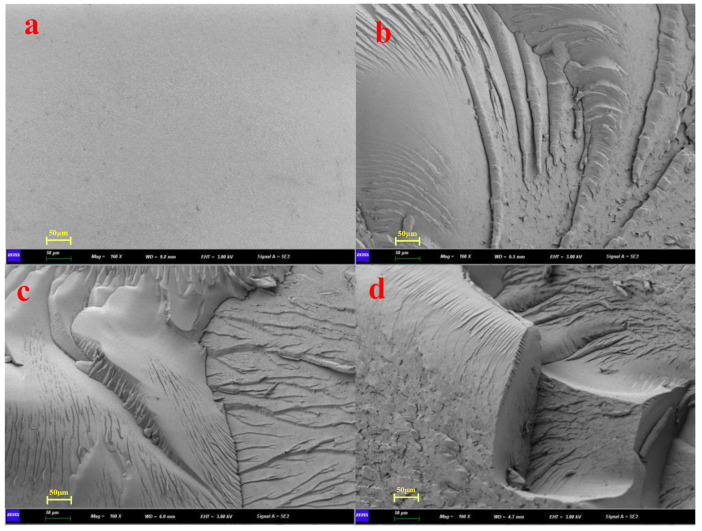
SEM micromorphology of the fracture surfaces of BMI-NH_2_. (**a**) BMI-0, (**b**) BMI-ODA, (**c**) BMI-FDA and (**d**) BMI-MT.

The bending fracture surface structures of BMI-NH_2_ samples are shown in Figure 5. Figure 5a shows the three-point bending of the pure BMI cured sample; its high cross-link density and strong rigidity yielded a relatively smooth fracture surface with a typical brittle fracture. This result showed that the toughness of BMI-0 was poor; the crack propagation was minimally hindered during the fracture process. However, the BMI cured samples modified using diamine could hinder crack propagation, toughened the BMI products, and increased the diffusion path during the fracture process to absorb more damage energy. Many ridge cracks and cliff-like shapes are shown in Figure 5b–d, which implied that the modified BMIs had typical ductile fracture characteristics. Based on the results of SEM, these characteristics identify the toughening effect and mechanical properties of diamine-modified BMI.

**Figure 6:**
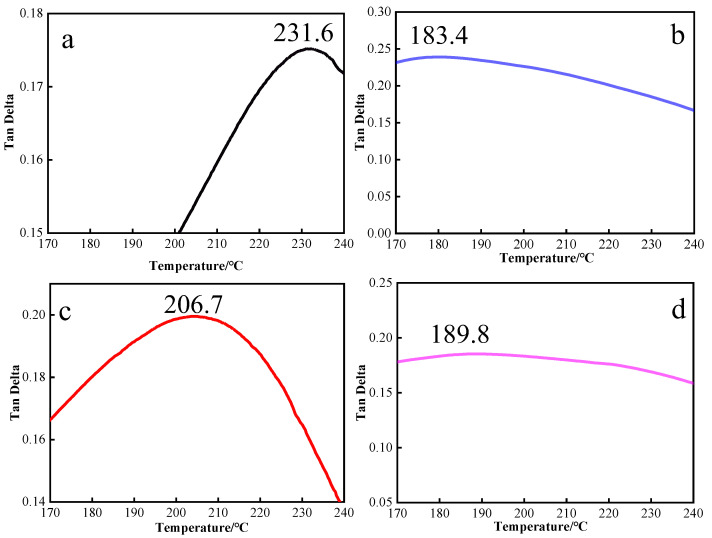
DMA curves of BMI-NH_2_. (**a**) BMI-0, (**b**) BMI-ODA, (**c**) BMI-FDA and (**d**) BMI-MT.

The glass transition temperatures (Tg) of samples were measured using DMA. Figure 6 shows the DMA curves of different types of diamine-modified BMI. According to the diagram, the Tg of the modified BMI samples generally decreased. Based on this, Flory’s free volume theory explains that when the free volume increased, the Tg of the cured compounds decreased. The polymer segments moved more easily due to the increase in temperature; that is, the segments’ transition from ‘freezing’ to ‘moving’ was more convenient. At the same time, the introduction of a branching structure increased the free volume of the system. When the temperature rose, the movement of the chain segments was not bound, resulting in a decrease in Tg. FDA has a rigid group, and ODA contains a flexible area, so that the thermal properties of the FDA sample decreased slightly. Although the introduction of free volume through the quaternary-branched structure had a negative effect on the thermal properties of BMI, the modified BMI still had excellent thermal properties in the working range of 180 ℃; the mechanical properties were greatly improved, while sacrificing the thermal properties. 

**Figure 7:**
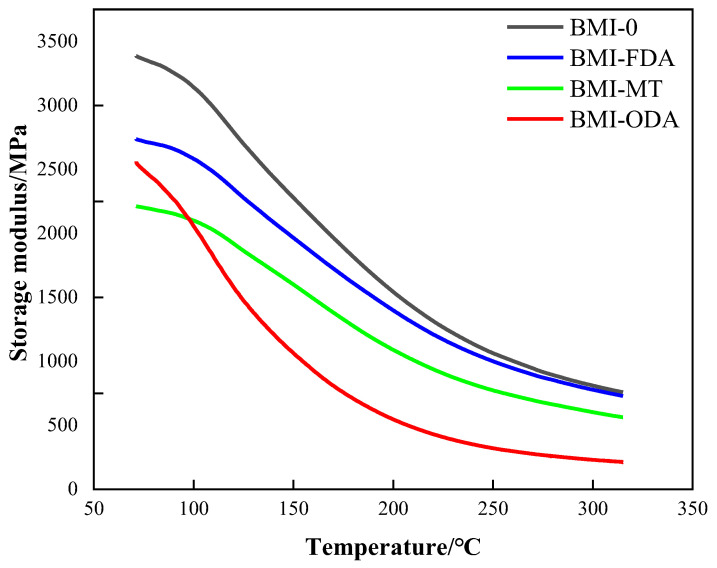
Storage modulus of BMI-NH2.

The storage modulus curves of BMI-NH2 are shown in Figure 7. It can be observed that the storage modulus of different samples decreased with an increase in temperature. This may have been due to the freezing state of the chain segment caused by a too low temperature; only the bond length and bond angle could slightly vibrate. The macroscopic segment was equivalent to freezing; as a result, the material maintained its original mechanical properties, and the initial storage modulus was larger. With an increase in temperature, the potential barrier of molecular motion decreased; at the same time, molecules had more thermal motion energy and the mobility of molecules increased, allowing more segments to move. Therefore, the storage modulus showed a downward trend. Figure 7 shows the storage modulus of samples with different types of diamines; the more obvious decrease was in the storage modulus of BMI-ODA samples. On the one hand, the branched structure introduced more free volume, so that the segments had more space; on the other hand, ODA itself contains flexible segments. Combining these two reasons, the enhancement of the movement ability of the chain segment reduced the storage modulus.

### 3.2. Insulate Performance

**Figure 8:**
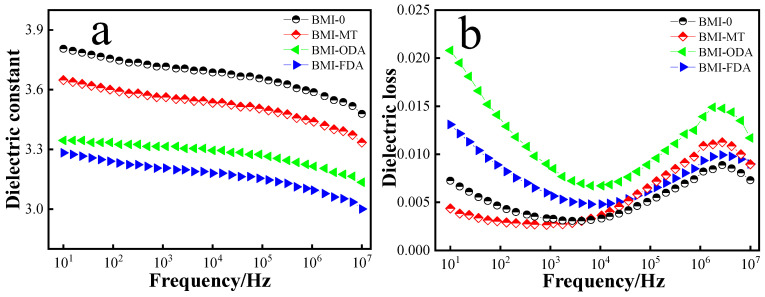
Dielectric property curves of BMI-NH_2_. (**a**) Dielectric property and (**b**) dielectric loss.

The dielectric constant curves of BMI-NH_2_ are shown in Figure 8a. Obviously, the dielectric constant of pure BMI and BMI samples decreased with increasing frequency. This is because when the electric field frequency was low, all types of polarization could keep up with a change in the electric field frequency. However, as the electric field frequency increased, the effective dipoles inside the sample were reduced. The branched structural resin was constructed using diamine, which brought free volume and decreased the content of polarization groups unit volume; the dielectric constant was reduced. In addition, due to the symmetrical molecular structure, the dielectric constant also decreased. As seen in Figure 8a, the dielectric constant of BMI-FDA was reduced further. This can be explained by the substantial free volume of functional groups of the FDA. It increased the distance of polymer molecules, reduced the inter-molecular force, and enhanced the movement ability of the molecular chain. However, the FDA had a rigid ring, which caused polarization difficulty. Thus, the dielectric constant was reduced.

The dielectric losses of the cured products with different structures of diamine are shown in Figure 8b; the variation trend of the dielectric loss with frequency of each sample was the same. The dielectric loss of the cure product reduced first, and then increased. For samples of BMI, dielectric loss was a priority with conductivity loss in the low-frequency electric field. There were conductivity losses and relaxation losses in the high-frequency electric field. In the low-frequency electric field, the minimum dielectric loss occurred with BMI-MT. The cure product reduced because of its branched structure, weakened intramolecular group interaction, and cross-linking density. As a result, the dielectric loss decreased. In the high-frequency electric field, the minimum dielectric loss occurred with BMI-FDA, with the exception of BMI-ODA. It brought a rigid ring into the system, making it harder to relax. As a result, the dielectric loss decreased.

**Figure 9:**
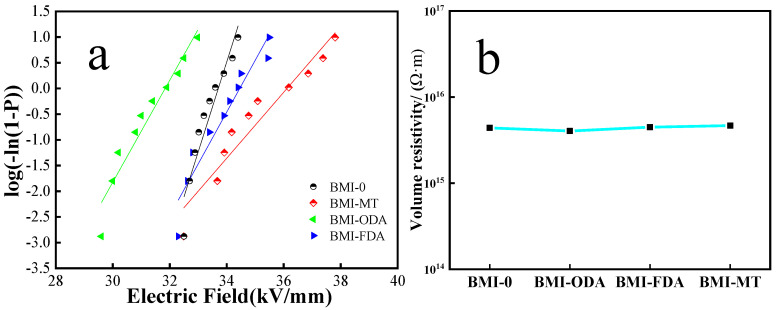
Insulate performance curves of BMI-NH_2_. (**a**) Breakdown strength and (**b**) volume resistivity.

Figure 9a is the breakdown field strength of BMI-NH_2_. The results indicate that the field strength of the modified BMI samples was ameliorated. Adding diamine to the BMI via Michael addition could avoid some of the internal defects of BMI and polish up the breakdown field strength of the BMI sample [36]. Diamine was added to the BMI via Michael addition to form branched structures, which facilitated the chain segment having a certain distance. The movement of the molecules was hindered, which increased the breakdown field strength. When the rigid segment (for example, FDA) was added to the structure of the BMI sample, the degree of stacking of molecular chains increased. Next, the number of free electrons accumulated unit volume decreased. The conductance property decreased and the breakdown field strength increased, relatively. BMI-ODA has a flexible group, which resulted in a reduction of breakdown field strength.

Figure 9b shows the volume resistivity of BMI-NH_2_; it can be intuitively seen that the volume resistivity of samples was basically unchanged. This was because the resin of the building branched structure multiplied the free volume of the system [31]. At the same time, the steric hindrance effect of the group resulted in a relatively compact structure. Under the action of both, when an external electric field was applied to the sample, the volume resistivity did not change. In addition, it can be observed that the different trends of the distinct structures of diamines were not the same. However, the sample of BMI-MT was slightly higher than BMI-ODA and BMI-FDA were, because it formed a tight and rigid structure that hindered the movement of electrons to some extent.

### 3.3. Thermal Properties

**Figure 10:**
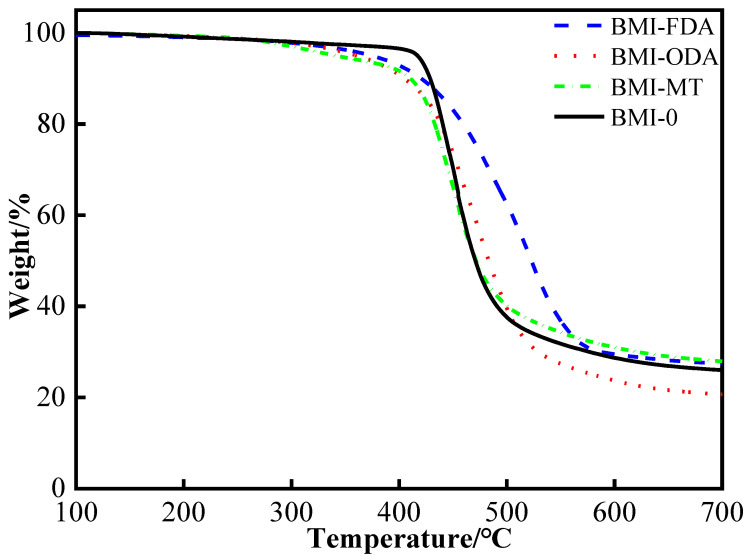
TG curves of BMI-NH_2_.

The thermogravimetry (TG) curves of BMI-NH_2_ in the N_2_ atmosphere are shown in Figure 10. It can be observed that the TG curve variation trends of BMI-0, BMI-ODA, BMI-FDA and BMI-MT were basically consistent until 300 ℃.; in the range of 300 ℃ to 400 ℃, the weight loss of modified BMI resin was greater than that of the pure BMI sample. This was because the nitrogen hydrogen bonds decomposed and the reagent used to modify the BMI contained a minor impurity. The stereo-hindrance effect caused a small number of sites in the system of BMI products that do not occur via the Michael addition reaction. After 400 ℃, it can be seen from the curves that the BMI-FDA decomposition temperature was higher than other samples, because there was a rigid group in the FDA, which could improve the BMI sample’s thermal stability. However, the flexible segment moved quickly at high temperature [37]; therefore, the TG decreased.

## 4. Conclusions

In this study, a branched structure via Michael addition was introduced to a BMI system to reinforce its toughness. The branched structure brought a large amount of free volume, which loosened the originally tightly arranged BMI resin.

➢ The flexural strength of BMI-ODA exhibited enhancement, and the maximum value of 189 MPa increased by 216%.
➢ The flexural modulus of BMI-FDA increased to 5.2 GPa.
➢ The fracture surface of BMI samples changedfrom brittle fracture to ductile fracture.
➢ Unfortunately, the glass transition temperatures of modified BMI resin were different degrees of decline. However, this did not affect its application in PCB.
➢ Owing to the branched structure, the electric dipole per unit volume was effectively reduced, and the dielectric property was degraded. The dielectric constant of BMI-FDA decreased to 3.0 in an electric field with a frequency of 10^7^ Hz, and the dielectric loss of BMI-ODA decreased to 0.0027 in an electric field with frequency between 10^2^ Hz and 10^3^ Hz.
➢ The breakdown field strength of BMI-MT was raised to 37.9 kV/mm^2^. The volume resistivity slightly improved, and the maximum value of BMI-MT was 4.66 × 10^15^ Ω·m.

Consequently, these results indicate that the modified BMI resin can meet the requirements of a wide variety of applications in high-frequency and low-dielectric resin substrates. The modified BMI resin with diamines of different structures with the best performance is suitable for various applications.

## Figures and Tables

**Table 1:** Formulations (Mass Ratio) of BMI-NH_2_.

Sample	BMI resin	ODA/g	FDA/g	MT/g
**BMI-0**	100			
**BMI-ODA**	100	13.9		
**BMI-FDA**	100		24.3	
**BMI-MT**	100			14.8

## Data Availability

The data presented in this study are available on request from the corresponding author.
